# On the Use of Manganese as an Adjuvant to Iron

**Published:** 1852-10

**Authors:** M. Petrequin


From the London Journal of Medicine.

*On the Use of Manganese as an adjuvant to Iron.* By M. PETREQUIN.

M. PETREQUIN quotes various authors to prove that manganese is a normal constituent of the animal and vegetable tissues; and believes that wherever iron is present in appreciable quantity, manganese co-exists with it. Hence iron alone will not always succeed in blood-diseases. M. Petrequin has observed many cases of chlorosis, which have resisted iron as obstinately as anæmia connected with cancer or organic degeneration. Other cases again, after deriving a certain amount of benefit from iron, remain stationary. Others again appear cured as iron, but the cure is not permanent. The remedy required in this case M. Petrequin finds to be manganese. He does not give it or iron alone, but combines them.

It is especially in *diseases of the blood* that ferro-manganic medicines are useful. They have a special action on the vascular apparatus, on the formation of the blood, and on the circulating fluid itself. They do not act as tonics or astringents; but as regenerators of the blood. They have succeeded admirably in anæmia following hæmorrhage, operations, polypi, metorrhagia, etc.; also in chlorosis attending puberty, which is a more common disease than is generally supposed, and occurs even in males. M. Petrequin has also frequently found the combinations of iron of benefit in the diseases of women at the critical period. He has often seen, in these subjects, *metorrhagia*, accompanied with an aspect of the surface which would lead to the suspicion of organic uterine disease: the hæmorrhage, however, was but a complication, and the patients, apparently in a hopeless state, have recovered under the use of ferro-manganic preparations, conjoined with tonics and ergotine.

In *amenorrhœa* and *dysmenorrhœa*, the patients often imagine that they require to be bled; but care must generally be taken not to comply with this request. M. Petrequin has more than once seen cases of amenorrhœa with severe chlorosis, in which it has not been desirable to hasten the appearance of the catamenia—the consequent loss of blood aggravating the disease. The general state of health must here be attended to. Œdema of the lower limbs sometimes occur in these cases; but it is a less severe complication than when it attends metorrhagia. It often disappears, as the patient recovers, under the use of iron and manganese.

These medicines are no less efficacious in the treatment of *anæmia* resulting from prolonged intermittent fevers, prolonged suppuration, strumous, syphilitic, or cancerous affections, phthisis, etc. Pills and the syrup of the iodide of manganese and iron are preferable in these cases.

In all these cases, the ferro-manganic preparations do not merely act on the stomach and nervous system, but they are absorbed, and assist in the formation of hæmatosine and new blood-globules, so as to restore the blood to its normal condition. Their effect in this way is greater than that of iron alone.

In the *functional affections of the heart* connected with chlorosis and anæmia, and which must not be mistaken for organic disease, a combination of iron and manganese with digitalis and other moderators of the heart's action is advantageous. The same remark applies to the *functional disorders of the lungs*, attending the same constitutional states.

*Disordered states of the nervous system* are intimately connected with those of the blood. He, as well as M. Gubian, has observed that iron is here better tolerated when combined with manganese. He has also seen benefit from the use of iron with manganese in many cases of *dyspepsia*, *gastralgia*, and *gastro-enteralgia*. Nervous affections of the digestive organs are often the result of chlorosis; and, where stomachics and chinchona have failed, iron has often been found (especially the carbonate, by some English physicians) to be of service. Gastrodynia complicating chlorosis has often yielded to the use of ferro-manganiferous water, and to pills of carbonate of iron and manganese.

In *nervous affections connected with exhaustion* from venereal excesses, onanism, rapid growth, &c., as well as in leucorrhœa, diabetis, &c., M. Petrequin has a high opinion of these medicines. He is continuing his researches on their action in certain cases of sterility from asthenia, and in some hyposthenic affections of the scalp, such as early baldness, alopecia, &c.

M. Petrequin has confined his observations to a limited number of the ferro-manganic preparations; and has made many observations before publishing the formulæ which he finds most useful. Having found, even at an early period, that the medicines were liable to adulteration, he has availed himself of the assistance of competent pharmaceutists. Since the publication of his first memoir. in 1849, these medicines have been extensively used in the South of France and in foreign countries.

The formulæ are few, and correspond to the preparations of iron generally used in France. They are: 1. *Pills* of carbonate of iron and manganese, or iodide; 2. *Lozenges* of lactate of iron and manganese; 3. *Syrups* of lactate or of iodide of iron and manganese; 4. Ferro-manganic *chocolate;* 5. *Effervescing solution* of iron and manganese.

It has been said that manganese not only preserves water, but purifies that which has undergone a change (Martin Lauzer). Ferro-manganic waters (of which there are many in France and other parts of the continent) can be preserved and carried to a distance;—which cannot generally be done with simple furruginous waters.

M. Petrequin commences by giving the powder of iron and manganese, with some vinous drink; he then administers two pills daily, one before breakfast and one before dinner, replacing them soon by lozenges. The syrups and chocolate complete the treatment. He gives the medicines at meal-time. The syrup he gives before breakfast, in doses of a tea-spoonful; and he finds it useful to administer directly after it some infusion of centaury, or of camomile flowers and orange.

Large doses are unnecessary and useless; for they are liable to produce irritation of the stomach and exhaustion of the nervous system; and the reparation of the blood is slow and progressive, and cannot, even were it desirable, be effected rapidly. Besides, the iron and manganese are not absorbed in any greater quantity, if large doses are given.

---

*Preparations of Manganese and Iron.*—M. Burin-Dubuisson of Lyons, who prepared most of the ferro-manganic combinations used by M. Petrequin, has published an interesting *brochure*, in which he gives the necessary details relating to the subject. The following formulæ are extracted from it.

*Powder for Effervescing Solution of Manganese and Iron.*—Take of coarsely powdered bicarbonate of soda 20 parts; tartaric acid, 25 parts; powdered sugar 53 parts; finely powdered sulphate of iron, 1½ parts; finely powdered sulphate of manganese, ¾ parts: mix carefully and keep in well stopped bottles. A teaspoonful is mixed with each glass of wine and water drunk during meal-time.

*Pills of Carbonate of Iron and Manganese.*—Take of pure crystallized sulphate of iron, 75 parts; pure crystallized sulphate of manganese, 25 parts; crystallized carbonate of soda, 120 parts; honey, 60 parts; water a sufficient quantity. Pills of 20 centigrammes (3 grains) are made; they keep easily, without becoming oxidized, in well-closed vessels. From two to four are given daily.

*Ferro-manganic Chocolate.*—One part of carbonate of iron and manganese is first mixed with four of sugar, and divided into large lozenges; of these, 100 parts (grammes) are mixed with 500 of chocolate paste, in the preparation of which 100 parts of sugar have been left out. This will make 800 lozenges, each of which contains about three centigrammes (nearly half a grain) of carbonate of iron and manganese. The chocolate decomposes the hydrated carbonate of manganese and iron of the saccharate into hydrated sesquioxide of iron and manganese; there is no metallic taste.

*Syrup of Lactate of Iron and Manganese.*—Take of lactate of iron and manganese, 4 parts; powdered sugar, 16 parts; rub together, and add of distilled water, 200 parts; dissolve rapidly, and pour into a mattrass over a water bath, containing 384 parts of broken sugar: filter the solution. This syrup contains about fifteen parts of lactate of iron and 5 of lactate of manganese in 3,000 parts. One or two spoonfuls are taken daily.

*Lozenges of Lactate of Iron and Manganese* are made by adding 20 parts of the lactate to 400 of fine sugar, with a sufficient quantity of water. The mass will make 840 lozenges; of which six or eight are taken daily.

*Syrup of Iodide of Iron and Manganese.*—M. Burin Dubuisson forms a solution of iodide of iron and manganese, in the proportion of one part by weight to two of water: the proportion of the salts is about three of iodide of iron to one of the iodide of manganese. Six parts of these are mixed with 294 of simple syrup; of this, M. Petrequin gives one or two tea-spoonfuls daily.

*Pills of Iodide of Iron and Manganese.*—Take of the officinal solution prepared by M. Burin Dubuisson, 15 parts (grammes); honey, 5 parts; some absorbent powder, 9½ parts. Divide into 100 pills. The honey and the solution are first mixed, and evaporated at first rapidly, then more slowly, to ten parts. Then add the powder, and divide the mass into four parts, which must be rolled in powder of iron reduced by hydrogen; each of these must then be divided on an iron plate into 25 pills, and again rolled in the iron powder. Finally, they are covered with a layer of tolu, according to M. Blanchard's process.

All these preparations must be made very carefully. M. Burin Dubuisson has ascertained that the commercial salts of manganese frequently contain copper, and even arsenic; he hence insists on the necessity of calcining the sulphate of manganese, twice, or more frequently, at a dark red heat, and of carefully testing the solution.

